# Supplementary material for: Assessment of genotype by environment and yield performance of tropical maize hybrids using stability statistics and graphical biplots
Source: PeerJ. 2024 Nov 29;12:e18624. doi: 10.7717/peerj.18624 (PMC11610465; doi:10.7717/peerj.18624)
Supplement: Supplemental Information 2 — *, ** and ***, significant at the 0.05, 0.01 and 0.001 probability levels, respectively; ns, non-significant; h2, broad-sense heritability; df, degrees of freedom; E01, Wonosari - Klaten; E02, Banguntapan - Tuban; E04, Jenu - Boyolali; E05, Ngronggot – Nganjuk; E06, Wonodadi – Blitar; E07, Plosoklaten – Kediri; E08, Bandar Kedungmulyo – Jombang; E09, Tumpang – Malang; E10, South Galesong – Talakar [file peerj-12-18624-s002.docx]

| **Environment** | **Mean Square** | | | **Mean grain**  **yield (t ha^-1^)** | **h^2^**  **(%)** |
| --- | --- | --- | --- | --- | --- |
|  | **Block** | **Genotype** | **Error** |  |  |
| E01 | 4.67*** | 2.58*** | 0.27 | 12.59 | 89.47 |
| E02 | 9.14*** | 1.83** | 0.48 | 12.24 | 73.67 |
| E03 | 0.06ns | 4.19*** | 0.24 | 10.47 | 94.38 |
| E04 | 0.01ns | 1.60* | 0.55 | 12.57 | 65.79 |
| E05 | 0.11ns | 3.22*** | 0.39 | 12.57 | 87.87 |
| E06 | 0.11ns | 5.50*** | 0.35 | 12.43 | 93.66 |
| E07 | 1.18ns | 3.97*** | 0.57 | 12.49 | 85.56 |
| E08 | 1.24* | 2.50*** | 0.30 | 12.48 | 88.05 |
| E09 | 2.09ns | 1.41ns | 0.82 | 12.51 | 41.49 |
| E10 | 3.10ns | 5.24** | 0.99 | 10.90 | 80.99 |
| df | 2 | 9 | 18 |  |  |
