# Supplementary material for: Assessment of genotype by environment and yield performance of tropical maize hybrids using stability statistics and graphical biplots
Source: PeerJ. 2024 Nov 29;12:e18624. doi: 10.7717/peerj.18624 (PMC11610465; doi:10.7717/peerj.18624)
Supplement: Supplemental Information 3 — Abbreviations: LSD, least significant difference; CV, coefficient of variation. [file peerj-12-18624-s003.docx]

| **Hybrids** | **E01** | **E02** | **E03** | **E04** | **E05** | **E06** | **E07** | **E08** | **E09** | **E10** | **Mean** |
| --- | --- | --- | --- | --- | --- | --- | --- | --- | --- | --- | --- |
| G01 | 13.29 | 13.22 | 10.42 | 13.95 | 13.79 | 13.87 | 13.80 | 13.76 | 13.87 | 11.92 | 13.19 |
| G02 | 13.53 | 12.72 | 10.50 | 12.23 | 12.87 | 13.18 | 12.08 | 12.02 | 12.80 | 13.21 | 12.51 |
| G03 | 13.84 | 13.33 | 12.01 | 13.02 | 13.03 | 14.11 | 13.42 | 12.60 | 12.28 | 9.14 | 12.68 |
| G04 | 12.92 | 11.71 | 11.12 | 12.36 | 13.19 | 11.85 | 13.77 | 13.37 | 12.64 | 11.90 | 12.48 |
| G05 | 11.96 | 12.62 | 10.72 | 12.82 | 13.71 | 13.47 | 12.00 | 13.50 | 13.05 | 11.50 | 12.53 |
| G06 | 11.51 | 12.05 | 8.45 | 11.78 | 11.28 | 12.00 | 12.15 | 12.50 | 11.41 | 10.92 | 11.40 |
| G07 | 12.72 | 12.45 | 12.05 | 12.81 | 13.37 | 13.43 | 13.03 | 12.49 | 11.96 | 10.59 | 12.49 |
| G08 | 10.83 | 10.79 | 8.74 | 11.25 | 11.97 | 11.33 | 10.85 | 10.74 | 11.92 | 11.10 | 10.95 |
| G09 | 12.64 | 11.75 | 10.57 | 12.80 | 11.43 | 10.82 | 13.23 | 12.12 | 12.36 | 9.32 | 11.70 |
| G10 | 12.69 | 11.72 | 10.11 | 12.68 | 11.08 | 10.27 | 10.58 | 11.67 | 12.76 | 9.41 | 11.30 |
| Mean | 12.59 | 12.24 | 10.47 | 12.57 | 12.57 | 12.43 | 12.49 | 12.48 | 12.51 | 10.90 | 12.12 |
| LSD 0.05 | 0.74 | 0.98 | 0.69 | 1.05 | 0.88 | 0.84 | 1.07 | 0.77 | 1.29 | 1.41 | 0.30 |
| CV (%) | 4.14 | 5.68 | 4.64 | 5.88 | 4.97 | 4.75 | 6.06 | 4.38 | 7.26 | 9.16 | 5.81 |
