# Supplementary material for: Assessment of genotype by environment and yield performance of tropical maize hybrids using stability statistics and graphical biplots
Source: PeerJ. 2024 Nov 29;12:e18624. doi: 10.7717/peerj.18624 (PMC11610465; doi:10.7717/peerj.18624)
Supplement: Supplemental Information 4 — The lower and upper limits represent the 95% confidence interval of prediction considering a two-tailed t-test. Abbreviations: LL, lower limit; UL, upper limit. The lower and upper limits represent the 95% confidence interval of prediction considering a two-tailed t-test. [file peerj-12-18624-s004.docx]

| **Rank** | **Hybrids** | **GY Mean** | **BLUPg** | **Predicted Mean** | **LL** | **UL** |
| --- | --- | --- | --- | --- | --- | --- |
| 1 | G01 | 13.19 | 0.94 | 13.07 | 12.54 | 13.59 |
| 2 | G03 | 12.68 | 0.49 | 12.62 | 12.09 | 13.14 |
| 3 | G05 | 12.53 | 0.36 | 12.49 | 11.96 | 13.01 |
| 4 | G02 | 12.51 | 0.35 | 12.47 | 11.94 | 13.00 |
| 5 | G07 | 12.49 | 0.32 | 12.45 | 11.92 | 12.97 |
| 6 | G04 | 12.48 | 0.32 | 12.44 | 11.92 | 12.97 |
| 7 | G09 | 11.70 | -0.37 | 11.75 | 11.23 | 12.28 |
| 8 | G06 | 11.40 | -0.64 | 11.48 | 10.96 | 12.01 |
| 9 | G10 | 11.30 | -0.73 | 11.39 | 10.86 | 11.92 |
| 10 | G08 | 10.95 | -1.04 | 11.08 | 10.56 | 11.61 |
