# Supplementary material for: Assessment of genotype by environment and yield performance of tropical maize hybrids using stability statistics and graphical biplots
Source: PeerJ. 2024 Nov 29;12:e18624. doi: 10.7717/peerj.18624 (PMC11610465; doi:10.7717/peerj.18624)
Supplement: Supplemental Information 5 — Abbreviations: GY, grain yield; CV, Coefficient of variance; bi, Regression coefficient; S2di, Deviation from regression; σ2i, Shukla’s stability variance; W2i, Wricke’s ecovalence; EV, Average of the squared eigenvector values; ASI, AMMI stability index; ASV, AMMI stability value; SIPC, Sum of the absolute value of the IPCA scores; MASV, Modified AMMI stability value; WAASB, Weighted average of absolute scores; Za, Absolute value of the relative contribution of IPCAs to the interaction; YSi, Kang’s rank sum; S(1, 2, 3, 6), Huehn’s and Nassar and Huehn’s statistics; NP(1-4), Thennarasu’s statistics. [file peerj-12-18624-s005.docx]

| **Statistic** | **Hybrids** | | | | | | | | | |
| --- | --- | --- | --- | --- | --- | --- | --- | --- | --- | --- |
|  | **G01** | **G02** | **G03** | **G04** | **G05** | **G06** | **G07** | **G08** | **G09** | **G10** |
| GY (t ha^-1^) | 13.19 | 12.51 | 12.68 | 12.48 | 12.53 | 11.40 | 12.49 | 10.95 | 11.70 | 11.30 |
| CV | 8.70 | 6.94 | 11.08 | 6.72 | 7.75 | 9.99 | 6.65 | 8.16 | 10.21 | 10.61 |
| *b_i_* | 1.43 | 0.56 | 1.28 | 0.74 | 0.98 | 1.22 | 0.74 | 0.81 | 1.17 | 1.05 |
| *S^2^_di_* | -0.06 | 0.47 | 0.95 | 0.26 | 0.25 | 0.29 | 0.24 | 0.29 | 0.52 | 0.72 |
| *σ^2^_i_* | 0.18 | 0.78 | 1.22 | 0.44 | 0.38 | 0.47 | 0.42 | 0.46 | 0.71 | 0.91 |
| *W^2^_i_* | 1.84 | 6.15 | 9.34 | 3.73 | 3.30 | 3.90 | 3.59 | 3.83 | 5.63 | 7.06 |
| EV | 0.05 | 0.14 | 0.13 | 0.12 | 0.11 | 0.11 | 0.07 | 0.04 | 0.08 | 0.15 |
| ASI | 0.10 | 0.37 | 0.54 | 0.09 | 0.19 | 0.21 | 0.26 | 0.35 | 0.38 | 0.31 |
| ASV | 0.46 | 1.68 | 2.48 | 0.39 | 0.88 | 0.95 | 1.17 | 1.59 | 1.76 | 1.44 |
| SIPC | 1.26 | 2.84 | 2.79 | 2.27 | 2.25 | 2.36 | 2.03 | 1.49 | 2.28 | 2.80 |
| MASV | 1.08 | 2.13 | 2.73 | 1.61 | 1.47 | 1.62 | 1.66 | 1.65 | 2.08 | 2.30 |
| Za | 0.13 | 0.31 | 0.39 | 0.20 | 0.22 | 0.23 | 0.24 | 0.21 | 0.31 | 0.32 |
| WAASB | 0.21 | 0.50 | 0.65 | 0.29 | 0.34 | 0.36 | 0.38 | 0.38 | 0.51 | 0.50 |
| YS_I_ | 13.00 | 1.00 | 3.00 | -1.00 | 6.00 | -8.00 | 0.00 | -10.00 | -6.00 | -9.00 |
| *S^(1)^* | 2.44 | 3.49 | 4.00 | 3.18 | 3.51 | 3.60 | 3.36 | 3.33 | 3.71 | 4.40 |
| *S^(2)^* | 4.27 | 8.54 | 11.29 | 7.12 | 8.71 | 9.07 | 8.23 | 9.07 | 9.79 | 13.73 |
| *S^(3)^* | 3.47 | 9.33 | 10.86 | 7.32 | 6.57 | 10.12 | 5.74 | 10.48 | 6.07 | 11.90 |
| *S^(6)^* | 1.30 | 3.48 | 3.14 | 2.92 | 2.29 | 5.29 | 2.32 | 5.30 | 3.16 | 4.97 |
| *NP^(1)^* | 1.60 | 2.30 | 2.80 | 2.10 | 2.20 | 2.60 | 2.10 | 2.20 | 2.50 | 3.20 |
| *NP^(2)^* | 1.07 | 0.42 | 1.12 | 0.53 | 0.63 | 0.35 | 0.42 | 0.24 | 0.36 | 0.40 |
| *NP^(3)^* | 0.93 | 0.57 | 0.89 | 0.54 | 0.70 | 0.38 | 0.57 | 0.33 | 0.44 | 0.45 |
| *NP^(4)^* | 0.03 | 0.02 | 0.01 | 0.01 | 0.00 | 0.01 | 0.01 | 0.00 | 0.02 | 0.02 |
