# Supplementary material for: Assessment of genotype by environment and yield performance of tropical maize hybrids using stability statistics and graphical biplots
Source: PeerJ. 2024 Nov 29;12:e18624. doi: 10.7717/peerj.18624 (PMC11610465; doi:10.7717/peerj.18624)
Supplement: Supplemental Information 6 [file peerj-12-18624-s006.docx]

| **Components** | **Eigenvalue** | **Proportion** | **Cumulative** |
| --- | --- | --- | --- |
| PC1 | 2.27E+00 | 42.20 | 42.20 |
| PC2 | 1.18E+00 | 21.90 | 64.10 |
| PC3 | 7.26E-01 | 13.50 | 77.60 |
| PC4 | 5.81E-01 | 10.80 | 88.40 |
| PC5 | 4.17E-01 | 7.80 | 96.20 |
| PC6 | 1.45E-01 | 2.70 | 98.90 |
| PC7 | 4.95E-02 | 0.90 | 99.80 |
| PC8 | 1.10E-02 | 0.20 | 100.00 |
| PC9 | 7.90E-04 | 0.00 | 100.00 |
| Total variance | 5.37E+00 | - | - |
